# Better PROMs and higher return-to-sport rate after modular bicompartmental knee arthroplasty than after total knee arthroplasty for medial and patellofemoral compartment osteoarthritis

**DOI:** 10.3389/fsurg.2022.1078866

**Published:** 2023-01-06

**Authors:** Wang Deng, Hongyi Shao, Hao Tang, Qiheng Tang, Zhaolun Wang, Dejin Yang, Yixin Zhou

**Affiliations:** Department of Orthopaedic Surgery, Beijing Jishuitan Hospital, Fourth Clinical College of Peking University, Beijing, China

**Keywords:** bicompartmental knee arthroplasty, total knee arthroplasty, medio-patellofemoral, osteoarthritis, return-to-sport rate

## Abstract

**Background:**

Theoretical advantages of bicompartmental knee arthroplasty (BKA) over total knee arthroplasty (TKA) for bicompartmental (medial combined with patellofemoral) osteoarthritis (OA) are still unclear. This study aimed to compare patient-reported outcome measures (PROMs) and return-to-sport (RTS) rate between modular BKA and TKA in early follow-up.

**Methods:**

Twenty-five consecutive modular BKA cases with a minimum 2-year follow-up were matched with 50 TKA cases at 1:2 ratio. Demographic data and preoperative functional scores, including the Western Ontario and McMaster Universities Osteoarthritis Index (WOMAC) and Knee Society Scores (KSSs), were analyzed to ensure comparability. Postoperative WOMAC score, KSS, range of motion (ROM), Forgotten Joint Score-12 (FJS-12), and RTS rates were compared. Operative time and blood loss were also analyzed.

**Results:**

Significant differences in the WOMAC-function (median 97.1 vs. 89.7, *p* < 0.001) and KSS-function (median 90.0 vs. 80.0, *p* = 0.003) scores were identified between the BKA and TKA groups. ROM was significantly greater in the BKA group than in the TKA group (median 125.0° vs. 120.0°, *p* = 0.004), in addition to the FJS-12 (median 89.6 vs. 53.1, *p* < 0.001). The overall RTS rate was significantly higher in the BKA group than in the TKA group (71.6% vs. 56.5%, *p* = 0.039). Operative time was significantly longer in the BKA group than in the TKA group (median 105.0 vs. 67.5 min, *p* < 0.001), but blood loss was similar (median 557.6 vs. 450.7 ml, *p* = 0.334).

**Conclusion:**

Modular BKA demonstrated better functional recovery, better joint perception, and higher RTS rate than TKA; thus, modular BKA can be a good alternative for bicompartmental OA.

## Introduction

Total knee arthroplasty (TKA), one of the most successful surgeries in the past 40 years, relieves pain and restores function in numerous patients with arthritis (1). However, as many as 20% of these patients are unsatisfied with their outcomes after TKA (2). Inherent in the design of TKA is the replacement of all three compartments of the knee in a single surgery, which inevitably compromises normal kinematics, function, gait, and proprioception (3, 4). Notably, primary osteoarthritis (OA) accounts for over 95% of all TKA procedures (1), while bicompartmental (medial combined with patellofemoral) OA comprises as much as 28%–50% of all TKA cases (5). This implies that the lateral compartment and cruciate ligaments are sacrificed in numerous patients when TKA is performed for bicompartmental OA. To overcome the intrinsic shortcomings of TKA, bicompartmental knee arthroplasty (BKA), a plausible alternative to TKA for bicompartmental OA, has attracted great interest from surgeons for several years, particularly for younger patients (6).

The primary advantage of BKA over TKA is less disruption of the normal knee with preserved lateral compartment and cruciate ligaments (7). The preservation of the anterior cruciate ligament (ACL) is paramount for the maintenance of the natural knee kinematic and proprioception (8–10), which may lead to better clinical outcomes after BKA over TKA. Moreover, as a less invasive procedure, BKA conforms to the philosophy of rapid recovery and may result in fewer perioperative complications (11). Revision surgery may also be easier. However, whether BKA can provide a better functional outcome over TKA has not been determined yet, and conflicting evidence has been reported (12–16). Moreover, the current goal of knee arthroplasty is not only to eliminate pain and restore joint functions but also to return to daily activities and, if possible, to sport (17). To the best of our knowledge, the return-to-sport (RTS) rate has not been compared between BKA and TKA.

Here, we performed a retrospective matched cohort study to compare patient-reported outcome measures (PROMs) and the RTS rate between patients who underwent modular BKA and those who underwent TKA in early follow-up.

## Methods

### Patient selection

With the approval of our institutional review board, 26 consecutive modular BKA cases performed by one surgeon between June 2015 and December 2018 at our hospital were identified. One patient died of a heart attack 1 year after the BKA procedure, with satisfactory knee outcomes. We matched the remaining 25 BKA cases with TKA performed by the same surgeon during the same period at a 1:2 ratio. Written informed consent has been obtained from all patients. The inclusion criteria for the BKA and TKA cases were as follows: (1) primary knee OA; (2) localized pain in the medial and anterior regions of the knee; and (3) radiographically localized OA features in the medial and patellofemoral compartments in the standard anteroposterior, lateral, and axial views of the knee (Kellgren-Lawrence level of the lateral compartment was not higher than level II). The exclusion criteria for patients were obvious deformities (flexion contracture >15°, a fixed varus or valgus deformity >15°), limited range of motion (ROM < 90°), and positive preoperative anterior/posterior drawer test and varus/valgus stress tests (18). We also excluded patients with any previous knee surgeries, including osteotomy and arthroscopy around the index knee. Patients with other diseases that may impair knee function, such as Parkinson's disease, stroke, heart failure, were also excluded.

To ensure comparability, we matched the BKA patient group with the TKA group by matching criteria including same gender, age within 3 years, body mass index (BMI) within 3 kg/m^2^, and operation date within 1 year. Finally, 50 TKA cases were included in the control group. Demographic data, preoperative Western Ontario and McMaster Universities Osteoarthritis Index (WOMAC) (ranging from 0 to 100, with higher scores indicating better results), American Knee Society Score (KSS), and ROM were analyzed for comparability. Preoperative coronal alignment was assessed using the hip-knee-ankle angle (HKA), which is the angle formed by the mechanical axis of the femur and the mechanical axis of the tibia on full-length films. A positive value indicates valgus alignment, whereas a negative value indicates varus alignment.

### Surgical technique

**Figure 1:**
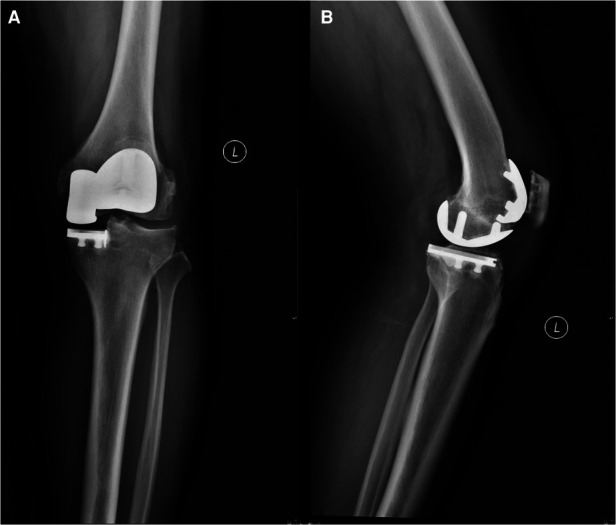
Postoperative knee radiography of patients who underwent BKA. (**A**) Anteroposterior view; (**B**) lateral view.

In all cases, spinal anesthesia was performed, and tourniquets were used. The medial parapatellar retinacular approach was performed after an anterior midline incision in both groups. We performed intraoperative assessment for all the scheduled BKA cases, including the status of cruciate ligaments and degeneration of lateral compartment. If the cruciate ligaments were assessed as ruptured or not functioning properly or full-thickness articular cartilage defects was observed in the lateral compartmental, the BKA procedure would be given up and changed into TKA procedure. A medial fixed-bearing unicompartmental implant (Zimmer, Warsaw, IN, USA) and an onlay patellofemoral implant (Zimmer, Warsaw, IN, USA) were combined and used as the modular BKA prosthesis (Figure 1). The Genesis II posterior-cruciate-retaining knee arthroplasty system (Smith & Nephew, Memphis, TN, USA) was used in the TKA group. Patellar resurfacing was performed in 11 cases in the BKA group and one case in the TKA group, and the remaining patients underwent osteophyte removal, smoothing of cartilage and patellar denervation according to the degenerative level of the patellar articular cartilage during surgery. In our practice, we commonly perform osteophyte removal, smoothing of cartilage of patella and patellar denervation in primary TKA or BKA instead of patellar resurfacing due to the concern about the risk of patellar fracture or implant loosening. If the degenerative level of the articular cartilage is severe, we performed patellar resurfacing no matter in TKA or BKA cases. No-thumb test was used to assess whether there is a tendency of dislocation and subluxation of the patella, and lateral patellar retinaculum was released appropriately if needed. For patients in which release of the lateral patellar retinaculum still could not reach the no-thumb test standards, we commonly trimmed the cartilage surface of the patella and reshaped the patella to make sure the patellar ridge was properly displaced medially and laterally to reach no-thumb test standards. All patients underwent a standard recovery plan with 3–5 days in the hospital for primary pain control and physical recovery. After being discharged from the hospital, all patients underwent the same at-home rehabilitation program.

### Outcome assessment

#### PROMs

The postoperative WOMAC scores and KSSs collected during the latest clinical visits were retrieved from the follow-up database. The KSS-knee was assessed by an experienced independent evaluator. The sub-score and total WOMAC were calculated, with a transformed score ranging from 0 (worst outcome) to 100 (best outcome). The Forgotten Joint Score-12 (FJS-12), which focuses on the joint perception after arthroplasty, was also used to measure the high performance level of the knee (19, 20).

#### Return-to-sport (RTS) rate

The patients were asked to list the sports they took part in “before the onset of restricting symptoms,” and if they had returned to these sports (21). We define the RTS as returning to the sport with similar or higher frequency per week or similar/longer duration per time. If they answered “returned,” the returned sports were recorded. If they answered, “not returned,” then the reasons were identified. The RTS rate was compared between the two groups. Any new sports performed after knee arthroplasty were also recorded.

#### Operative time and blood loss

Operative time was retrieved from the medical records and compared between the two groups. Blood loss was calculated based on the height, weight, sex, drop in hemoglobin level, and blood transfusion following a previously reported formula (22).

### Statistics analysis

Continuous variables were expressed as mean with range or median with interquartile range (IQR) and were compared using Student's *t*-test or Mann-Whitney test according to the Shapiro-Wilk test of normality. Categorical variables were compared between the two groups using the chi-square test or Fisher's exact test. SPSS version 23.0 (IBM, Armonk, New York, USA) was used to perform statistical analyses, with *p* < 0.05 as the statistically significant threshold.

## Results

### Preoperative baseline information

Results of preoperative demographic data, functional scores (WOMAC scores, KSSs), and alignment demonstrated the comparability of the BKA and TKA groups (Table 1). There was no significant difference between the preoperative ROM (*p* = 0.890), and the two groups showed similar coronal alignment, as assessed by the HKA angle (*p* = 0.291).

**Table 1:** Comparison of demographic and preoperative data between the BKA and TKA groups.

Preoperative parameters	BKA (*n* = 25)	TKA (*n* = 50)	*p*
Age, years^a^	60.0 ± 8.0	62.2 ± 6.1	0.198
Male^b^	14 (56.0%)	28 (56.0%)	1.000
BMI, kg/m^2a^	26.1 ± 3.0	26.5 ± 2.5	0.548
WOMAC^a^	54.9 ± 12.2	58.6 ± 12.6	0.223
WOMAC-pain^c^	55.0 (50.0,70.0)	60.0 (50.0,70.0)	0.170
WOMAC-stiffness^c^	50.0 (50.0,75.0)	62.5 (50.0, 62.5)	0.191
WOMAC-function^a^	54.18 ± 13.51	58.29 ± 14.49	0.239
KSS-knee^c^	59.0 (44.5, 66.5)	61.0 (50.5, 68.25)	0.336
KSS-function^c^	50.0 (50.0, 60.0)	50.0 (50.0, 60.0)	0.807
ROM (degree)^c^	120.0 (90.0,120.0)	117.5 (100.0, 125.0)	0.890
HKA (degree)^c^^,^^d^	−6.8 (−7.8, −4.5)	−7.1 (−9.5 −4.9)	0.291

BKA, bicompartmental knee arthroplasty; TKA, total knee arthroplasty; BMI, body mass index; WOMAC, Western Ontario and McMaster Universities Osteoarthritis Index; KSS, Knee Society Score; ROM, range of motion; HKA, the hip-knee-ankle angle.

^a^ Mean value with standard deviation (SD)and compared by Student's *T*-test.

^b^ Compared by Chi-square test.

^c^ Median value with interquartile range and compared by Mann-Whitney test.

^d^ Negative value indicates the varus alignment.

### PROMs

The median follow-up time was 4.8 years in the BKA group and 4.6 years in the TKA group, respectively. After a similar follow-up period, no revisions occurred in either group. The WOMAC score in the BKA group was better than that in the TKA group (*p* < 0.001, Table 2); the same was true for the WOMAC-function score (*p* < 0.001). The KSS-function score in the BKA group was also significantly better than that in the TKA group (*p* = 0.003). In the BKA group, the ROM was greater (*p* = 0.004) and the FJS-12 was higher (*p* < 0.001). No cases in either group demonstrated component loosening, and no progression of OA severity was identified radiographically in the BKA group. Comparison between BKA with or without patellar resurfacing indicated that there was no difference in terms of postoperative WOMAC score (*p* = 0.647), KSS-function score (*p* = 0.244) and FJS-12 (*p* = 1.000).

**Table 2:** Comparison of postoperative data between the BKA and TKA groups.

Postoperative parameters^a^	BKA	TKA	*p*
Follow up (year)	4.8 (2.3, 5.2)	4.6 (2.3 5.6)	0.450
WOMAC	97.9 (92.7, 100.0)	91.7 (88.3, 95.8)	**<0.001**
WOMAC-pain	95.0 (100.0, 100.0)	100.0 (100.0, 100.0)	0.509
WOMAC-stiffness	100.0 (100.0, 100.0)	93.8 (75.0, 100.0)	**0.003**
WOMAC-function	97.1 (91.9, 100.0)	89.7 (85.3, 94.1)	**<0.001**
KSS-knee	100.0 (91.5, 100.0)	95.0 (88.8, 99.3)	**0.045**
KSS-function	90.0 (80.0, 100.0)	80.0 (70.0, 90.0)	**0.003**
FJS-12	89.6 (59.4, 96.9)	53.1 (43.2, 73.4)	**<0.001**
ROM (degree)	125.0 (120.0, 130.0)	120.0 (110.0, 125.0)	**0.004**
HKA (degree)^b^	−2.1 (−5.2, −1.3)	−1.8 (−3.8, 0.1)	0.291

BKA, bicompartmental knee arthroplasty; TKA, total knee arthroplasty; WOMAC, Western Ontario and McMaster Universities Osteoarthritis Index; KSS, Knee Society Score; FJS-12, Forgotten joint score-12; ROM, range of motion; HKA, the hip-knee-ankle angle. The bold values indicated the *p* value was <0.05.

^a^ Median value with interquartile range and compared by Mann-Whitney test.

^b^ Negative value indicates the varus alignment.

### Return-to-sport rate

**Figure 2:**
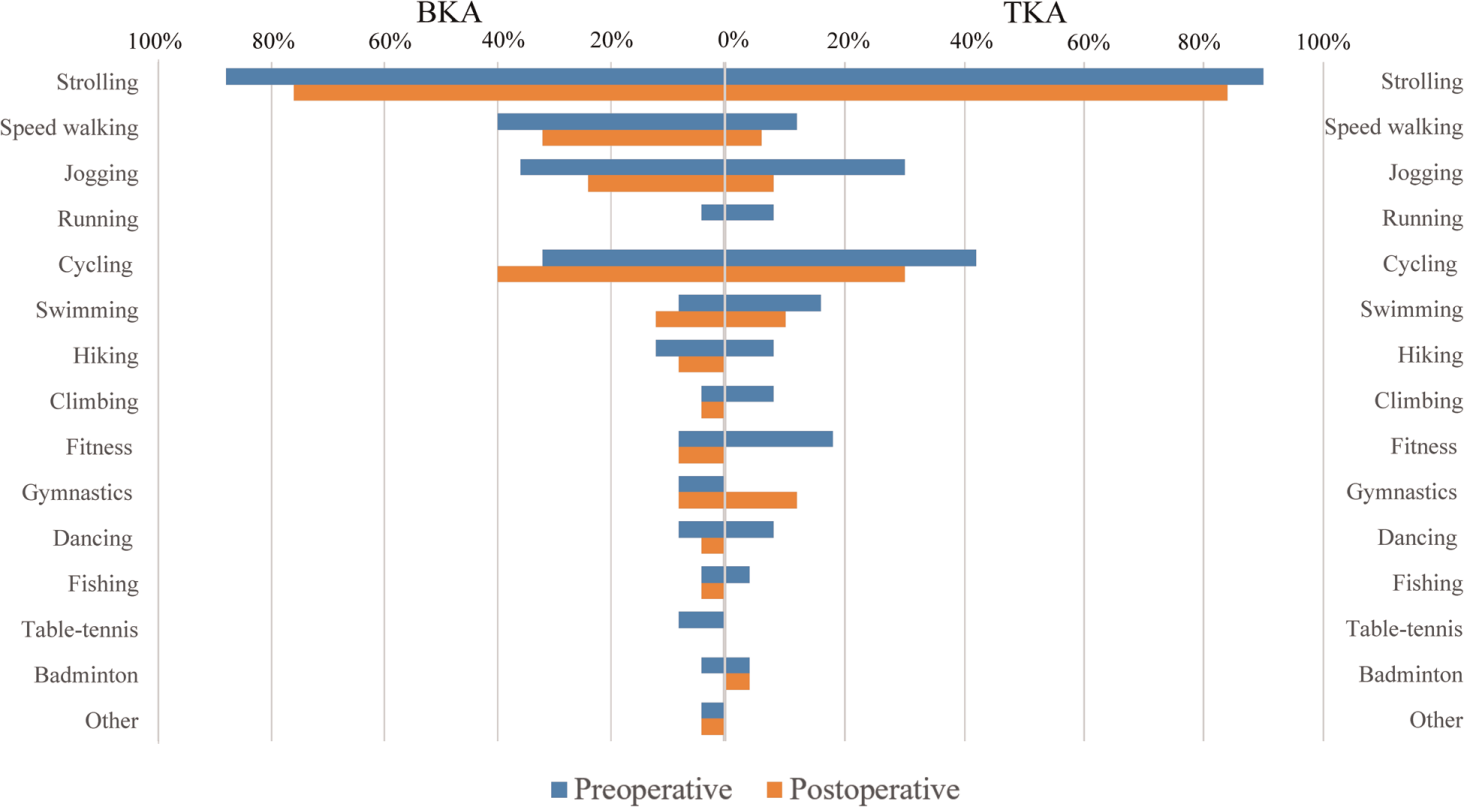
Preoperative and postoperative sports activities between the BKA and TKA groups.

**Figure 3:**
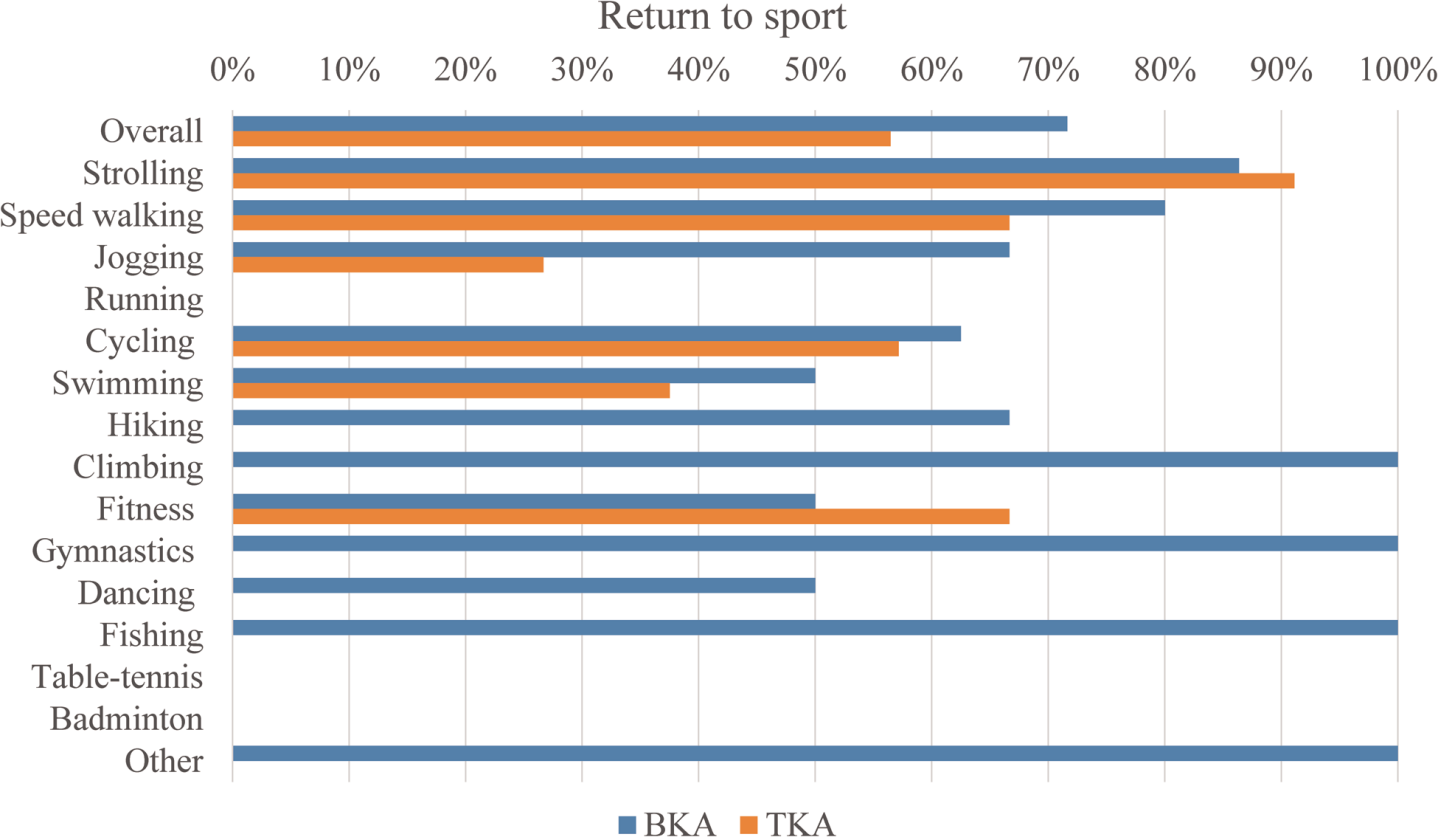
Comparison of return-to-sport rates between the BKA and TKA groups.

Preoperative and postoperative sports activities were identified in the two groups (Figure 2). The overall RTS rate was higher in the BKA group (71.64% vs. 56.45%, *p* = 0.039) (Figure 3). Protection of knee was the main reason for the failure of RTS in both groups, whereas the proportions of different reasons in the two groups differed (*p* = 0.028) (Table 3). Limited function took much higher proportion in TKA group (31.5%) than that in BKA group (5.3%) (*p* = 0.008). There was no difference detected between BKA with or without patellar resurfacing in terms of RTS rate (66.7% vs. 75.0%, *p* = 0.458).

**Table 3:** The reasons for failed return-to-sport (RTS) between the BKA and TKA groups.

Reasons for failed RTS^a^	Failed RTS in BKA (*n* = 19^b^)	Failed RTS in TKA (*n* = 54^b^)	*p*
Protection of knee	14 (73.7%)	18 (33.3%)	**0.028** ^a^ ^,^ ^c^
Pain after sport	3 (15.8%)	10 (18.5%)	
Limited function	1 (5.3%)	17 (31.5%)	
Comorbidity	1 (5.3%)	7 (13.0%)	
Other	0 (0.0%)	2 (3.7%)	

BKA, bicompartmental knee arthroplasty; TKA, total knee arthroplasty. The bold values indicated the *p* value was <0.05.

^a^ Fisher exact test between all the different reasons.

^b^ The number of sports activities failed to return.

^c^ Comparison between protection and limited function (*p* = 0.008 < 0.01; Bonferroni correction).

### Operative time and blood loss

The operative time for the BKA group was significantly longer than that for the TKA group (median 105.0 vs. 67.5 min, *p* < 0.001) (Table 4). No difference was observed in the calculated blood loss between the two groups (*p* = 0.334). Only one transfusion amounting to 800 ml of packed red blood cells occurred in the TKA group, which was for a case of simultaneous bilateral TKA. No complications, including infection, thrombosis, or anesthesia-related complications, occurred in either group.

**Table 4:** Comparison of operative time and blood loss between the BKA and TKA groups.

Parameters^a^	BKA	TKA	*p*
Operative time (min)	105.0 (90.0, 125.0)	67.5 (60.0, 90.0)	**<0.001**
Calculated blood loss (ml)	557.6 (384.2, 688.9)	450.7 (359.0,683.3)	0.334

BKA, bicompartmental knee arthroplasty; TKA, total knee arthroplasty. The bold values indicated the *p* value was <0.05.

^a^ Median value with interquartile range and compared by Mann-Whitney test.

## Discussion

Our study showed that modular BKA may lead to improved PROMs and a higher RTS rate than TKA for bicompartmental OA. Moreover, better ROM and joint perception were observed after modular BKA than after TKA. Although the surgical time was longer in the modular BKA procedure than in TKA, blood loss and perioperative complications were similar.

Our results are consistent with those of Parratte's study (14). Modular BKA showed better functional performance in both our study and Parratte's report in terms of KSSs. Furthermore, patients in the BKA group showed improved functions, as assessed by the WOMAC-function score in this study and the KOOS sports score in Parratte's study. Better functional recovery and mechanical performance after BKA than after TKA is also supported by previous kinetic analyses (23–25). In these models, knee mechanics during weight-bearing flexions, stand-to-sit movement, and step ascent and descent after BKA were more similar to those in healthy controls than after TKA (23, 24). Quadriceps strength after BKA was comparable to that of healthy controls and nearly 20% greater than that after TKA (25).

In contrast to our study, some previous studies demonstrated that BKA and TKA had similar functional scores and comparable functional performance (12, 15, 16). This could be due to various factors; implant choice was the first reason to be taken into account. In monolithic BKA, poor results and a high revision rate may be due to the limited size choice and variability in morphology of the distal femur (13). Given that it is technically challenging to achieve optimal sizing, position, and balancing for the two compartments, using a monolithic implant is not advisable. In modular BKA, inconsistency of the clinical outcomes may also be due to the different implants used. Different designs of the patellar components in our study (onlay-design) compared to those in other studies (inlay-design) may be the reason for the conflicting results (12, 15, 16, 22). The onlay-design components are recommended as they have demonstrated better functional outcomes, improved patellar tracking, and lower failure rate (26). Therefore, defects in the former implants, instead of the BKA procedure itself, may have led to the underestimation of functional improvement after BKA. New generation modular implants for BKA have shown excellent functional results (27, 28). Another reason for the inconsistencies across studies may be discrepancy in the inclusion criteria for modular BKA in these studies. The consistency of the BKA group in Biazzo's study was greatly impaired because of the inclusion of the lateral compartment combined with patellofemoral arthroplasty. In addition, the implants used were diverse (29). The third possible factor is the incomparability of preoperative parameters, such as significantly different preoperative ROM in Shah's study or mean age in Tan's report, which may confound the factors analyzed in these studies (30, 31).

Aside from clinical function, differences in subjective feeling after BKA and TKA is of interest; “forgotten status” was considered the ultimate goal of arthroplasty (19). This status may indicate improved kinematics and proprioception, which plays an important role in function and the avoidance of falling after arthroplasty (4). The sensor role of cruciate ligaments has been proven and absence of ACL will lead to reduced proprioception and kinesthesia, abnormal patterns of muscle activity (32–34). In theory, more mechanoreceptors are preserved in BKA. However, no direct studies comparing proprioception after BKA and TKA have been performed. Three BKA cases in our study achieved the “fully forgotten” knee (FJS-12 score = 100), which is lower than other reported data where “fully forgotten” status was achieved in 20 out of 34 patients (14). However, 12 of the 25 cases in the BKA group in our study scored >90 points on the FJS-12, and the rate was significantly higher than that in the TKA group (2/50), demonstrating the advantage of modular BKA over TKA in terms of joint awareness. This may be due to the modular BKA better preserving soft tissue, ACL, and stronger quadriceps strength, which have been reported to improve postoperative stability and joint unawareness of the knee (4, 35).

Another important finding of this study was that the RTS rate was higher after BKA than after TKA. In a recent meta-analysis, better RTS after unicompartmental knee arthroplasty (UKA) (75% to over 100%) was observed than after TKA (36%–89%) (36). As an important constituent of BKA, patellofemoral arthroplasty (PFA) also showed a high RTS rate. Return to previous preferred activity after PFA was reported by 72.2% of patients, and 52.8% reported returning to the same activity level or higher (37). Based our data and previous publications, we believe BKA, the combination of UKA and PFA, did not lead to a lower RTS rate than UKA or PFA alone. This confirmed the minimal invasiveness of BKA. We also found that the reasons for failure of RTS were different between the two groups. Limited function caused 31.5% of the failure to RTS in the TKA group, while the counterpart in the BKA group was only 5.3%, confirming improved functional recovery after BKA compared to TKA. The RTS restricted by protection of the knee in the BKA group was high and could be further reduced under appropriate encouragement and sports instruction.

Admittedly, the mean operative time for modular BKA procedures was significantly longer than that for TKA. This makes sense considering the complexity of the modular BKA procedure and the proficiency of the TKA procedure for surgeons who have performed a large number of TKA cases. This result has also been confirmed by other studies (29, 30). Although the surgeon is familiar with both unicompartmental knee and patellofemoral arthroplasty, a learning curve still exists for modular BKA as it is a technically demanding intervention, and operative time may decrease with practice. Although the operative time for modular BKA was longer, there was no increased risk of perioperative complications or increased blood loss.

Our study has several limitations. The first is the lower proportion of patients in the TKA group underwent patellar resurfacing. However, all the cases in both groups went through patellar denervation and a recent network meta-analysis which included 18 randomized controlled trials (RCT) or quasi-experimental studies supported that there was no difference in pain score nor functional performance after TKA with patellar denervation or patellar resurfacing (38). Moreover, a recent meta-analysis of RCTs showed that patellar resurfacing itself only increased by 1.67 in the KSS-function score in TKA, which is too small to be clinically significant (39). Our data also confirmed there was no difference in terms of postoperative scores and return-to-sport rate between BKA cases with or without patellar resurfacing. Based on these studies and data, this limitation may not impair the comparability between the two groups. Second, most of the sports in the included patients were of low to medium impact level, which did not reflect the possible difference in high-impact sports. To the best of our knowledge, this is the first study to compare the RTS between BKA and TKA procedures, and we found a difference in RTS between the two groups. At last, the sample size in this early follow-up study was comparatively small. Prospective comparative studies with larger sample sizes and long-term follow-up should be conducted to further confirm the findings of the current study.

## Conclusion

BKA demonstrated better PROMs and higher RTS rates than TKA, indicating that BKA is a good surgical alternative for the treatment of bicompartmental OA.

## Data Availability

The raw data supporting the conclusions of this article will be made available by the authors, without undue reservation.

## References

[1] Carr AJ Robertsson O Graves S Price AJ Arden NK Judge A Knee replacement. Lancet. (2012) 379(9823):1331–40. 10.1016/S0140-6736(11)60752-6 22398175

[2] Bourne RB Chesworth BM Davis AM Mahomed NN Charron KD. Patient satisfaction after total knee arthroplasty: who is satisfied and who is not? Clin Orthop Relat Res. (2010) 468(1):57–63. 10.1007/s11999-009-1119-9 19844772 PMC2795819

[3] Standifird TW Saxton AM Coe DP Cates HE Reinbolt JA Zhang S. Influence of total knee arthroplasty on gait mechanics of the replaced and non-replaced limb during stair negotiation. J Arthroplasty. (2016) 31(1):278–83. 10.1016/j.arth.2015.06.052 26231075

[4] Wodowski AJ Swigler CW Liu H Nord KM Toy PC Mihalko WM. Proprioception and knee arthroplasty: a literature review. Orthop Clin North Am. (2016) 47(2):301–9. 10.1016/j.ocl.2015.09.005 26772938

[5] Heekin RD Fokin AA. Incidence of bicompartmental osteoarthritis in patients undergoing total and unicompartmental knee arthroplasty: is the time ripe for a less radical treatment? J Knee Surg. (2014) 27(1):77–81. 10.1055/s-0033-1349401 23873317

[6] Sabatini L Giachino M Risitano S Atzori F. Bicompartmental knee arthroplasty. Ann Transl Med. (2016) 4(1):5. 10.3978/j.issn.2305-5839.2015.12.24 26855941 PMC4716949

[7] Garner A van Arkel RJ Cobb J. Classification of combined partial knee arthroplasty. Bone Joint J. (2019) 101-B(8):922–8. 10.1302/0301-620X.101B8.BJJ-2019-0125.R1 31362558 PMC6681677

[8] Johnson AJ Howell SM Costa CR Mont MA. The ACL in the arthritic knee: how often is it present and can preoperative tests predict its presence? Clin Orthop Relat Res. (2013) 471(1):181–8. 10.1007/s11999-012-2505-2 22864617 PMC3528932

[9] Banks SA Fregly BJ Boniforti F Reinschmidt C Romagnoli S. Comparing in vivo kinematics of unicondylar and bi-unicondylar knee replacements. Knee Surg Sports Traumatol Arthrosc. (2005) 13(7):551–6. 10.1007/s00167-004-0565-x 15660274

[10] Heyse TJ El-Zayat BF De Corte R Scheys L Chevalier Y Fuchs-Winkelmann S Biomechanics of medial unicondylar in combination with patellofemoral knee arthroplasty. Knee. (2014) 21(S1):S3–9. 10.1016/S0968-0160(14)50002-6 25382365

[11] Thienpont E Price A. Bicompartmental knee arthroplasty of the patellofemoral and medial compartments. Knee Surg Sports Traumatol Arthrosc. (2013) 21(11):2523–31. 10.1007/s00167-012-2303-0 23184084

[12] Uluyardimci E Isik C Tahta M Emre F Cepni S Oltulu I. The combination of inlay patellofemoral arthroplasty and medial unicompartmental knee arthroplasty versus total knee arthroplasty for mediopatellofemoral osteoarthritis: a comparison of mid-term outcomes. J Arthroplasty. (2019) 34(11):2614–9. 10.1016/j.arth.2019.06.043 31320188

[13] Amit P Singh N Soni A Bowman NK Maden M. Systematic review of modular bicompartmental knee arthroplasty for medio-patellofemoral osteoarthritis. J Arthroplasty. (2020) 35(3):893–9. 10.1016/j.arth.2019.09.042 31676175

[14] Parratte S Ollivier M Opsomer G Lunebourg A Argenson JN Thienpont E. Is knee function better with contemporary modular bicompartmental arthroplasty compared to total knee arthroplasty? Short-term outcomes of a prospective matched study including 68 cases. Orthop Traumatol Surg Res. (2015) 101(5):547–52. 10.1016/j.otsr.2015.03.019 26047754

[15] Goh JKM Chen JY Yeo NEM Liow MHL Chia SL Yeo SJ. Ten year outcomes for the prospective randomised trial comparing unlinked, modular bicompartmental knee arthroplasty and total knee arthroplasty. Knee. (2020) 27(6):1914–22. 10.1016/j.knee.2020.08.013 33221689

[16] Schrednitzki D Beier A Marx A Halder AM. No major functional benefit after bicompartmental knee arthroplasty compared to total knee arthroplasty at 5-year follow-up. J Arthroplasty. (2020) 35(12):3587–93. 10.1016/j.arth.2020.07.003 32739080

[17] Canovas F Dagneaux L. Quality of life after total knee arthroplasty. Orthop Traumatol Surg Res. (2018) 104(S1):S41–S6. 10.1016/j.otsr.2017.04.017 29183821

[18] Berger Y Ftaita S Thienpont E. Does medial patellofemoral osteoarthritis influence outcome scores and risk of revision after fixed-bearing unicompartmental knee arthroplasty? Clin Orthop Relat Res. (2019) 477(9):2041–7. 10.1097/CORR.0000000000000738 31140980 PMC7000094

[19] Behrend H Giesinger K Giesinger JM Kuster MS. The “forgotten joint” as the ultimate goal in joint arthroplasty: validation of a new patient-reported outcome measure. J Arthroplasty. (2012) 27(3):430–6. 10.1016/j.arth.2011.06.035 22000572

[20] Cao S Liu N Han W Zi Y Peng F Li L Simplified Chinese version of the forgotten joint score (FJS) for patients who underwent joint arthroplasty: cross-cultural adaptation and validation. J Orthop Surg Res. (2017) 12(1):6. 10.1186/s13018-016-0508-5 28088227 PMC5237477

[21] Ho JC Stitzlein RN Green CJ Stoner T Froimson MI. Return to sports activity following UKA and TKA. J Knee Surg. (2016) 29(3):254–9. 10.1055/s-0035-1551835 26166426

[22] Yeo NE Chen JY Yew A Chia SL Lo NN Yeo SJ. Prospective randomised trial comparing unlinked, modular bicompartmental knee arthroplasty and total knee arthroplasty: a five years follow-up. Knee. (2015) 22(4):321–7. 10.1016/j.knee.2015.04.007 25956739

[23] Wang H Dugan E Frame J Rolston L. Gait analysis after bi-compartmental knee replacement. Clin Biomech. (2009) 24(9):751–4. 10.1016/j.clinbiomech.2009.07.014 19695749

[24] Leffler J Scheys L Plante-Bordeneuve T Callewaert B Labey L Bellemans J Joint kinematics following bi-compartmental knee replacement during daily life motor tasks. Gait Posture. (2012) 36(3):454–60. 10.1016/j.gaitpost.2012.04.008 22748470

[25] Wang H Foster J Franksen N Estes J Rolston L. Gait analysis of patients with an off-the-shelf total knee replacement versus customized bi-compartmental knee replacement. Int Orthop. (2018) 42(4):805–10. 10.1007/s00264-017-3622-z 28868567

[26] Lustig S Magnussen RA Dahm DL Parker D. Patellofemoral arthroplasty, where are we today? Knee Surg Sports Traumatol Arthrosc. (2012) 20(7):1216–26. 10.1007/s00167-012-1948-z 22407183

[27] Rossi SMP Perticarini L Clocchiatti S Ghiara M Benazzo F. Mid- to long-term follow-up of combined small implants. Bone Joint J. (2021) 103(5):840–5. 10.1302/0301-620X.103B5.BJJ-2020-0720.R3 33934658

[28] Romagnoli S Marullo M. Mid-term clinical, functional, and radiographic outcomes of 105 gender-specific patellofemoral arthroplasties, with or without the association of medial unicompartmental knee arthroplasty. J Arthroplasty. (2018) 33(3):688–95. 10.1016/j.arth.2017.10.019 29129614

[29] Biazzo A Silvestrini F Manzotti A Confalonieri N. Bicompartmental (uni plus patellofemoral) versus total knee arthroplasty: a match-paired study. Musculoskelet Surg. (2019) 103(1):63–8. 10.1007/s12306-018-0540-1 29654550

[30] Shah SM Dutton AQ Liang S Dasde S. Bicompartmental versus total knee arthroplasty for medio-patellofemoral osteoarthritis: a comparison of early clinical and functional outcomes. J Knee Surg. (2013) 26(6):411–6. 10.1055/s-0033-1343612 23575564

[31] Tan SM Dutton AQ Bea KC Kumar VP. Bicompartmental versus total knee arthroplasty for medial and patellofemoral osteoarthritis. J Orthop Surg. (2013) 21(3):281–4. 10.1177/230949901302100303 24366784

[32] Johansson H Sjolander P Sojka P. A sensory role for the cruciate ligaments. Clin Orthop Relat Res. (1991) 268:161–78. 2060205

[33] Fremerey RW Lobenhoffer P Zeichen J Skutek M Bosch U Tscherne H. Proprioception after rehabilitation and reconstruction in knees with deficiency of the anterior cruciate ligament: a prospective, longitudinal study. J Bone Joint Surg Br. (2000) 82(6):801–6. 10.1302/0301-620X.82B6.0820801 10990300

[34] Chmielewski TL Stackhouse S Axe MJ Snyder-Mackler L. A prospective analysis of incidence and severity of quadriceps inhibition in a consecutive sample of 100 patients with complete acute anterior cruciate ligament rupture. J Orthop Res. (2004) 22(5):925–30. 10.1016/j.orthres.2004.01.007 15304261

[35] Hiyama Y Wada O Nakakita S Mizuno K. Joint awareness after total knee arthroplasty is affected by pain and quadriceps strength. Orthop Traumatol Surg Res. (2016) 102(4):435–9. 10.1016/j.otsr.2016.02.007 27052936

[36] Witjes S Gouttebarge V Kuijer PP van Geenen RC Poolman RW Kerkhoffs GM. Return to sports and physical activity after total and unicondylar knee arthroplasty: a systematic review and meta-analysis. Sports Med. (2016) 46(2):269–92. 10.1007/s40279-015-0421-9 26744336 PMC4728176

[37] Shubin Stein BE Brady JM Grawe B Tuakli-Wosornu Y Nguyen JT Wolfe E Return to activities after patellofemoral arthroplasty. Am J Orthop. (2017) 46(6):E353–E7. 29309448

[38] Arirachakaran A Sangkaew C Kongtharvonskul J. Patellofemoral resurfacing and patellar denervation in primary total knee arthroplasty. Knee Surg Sports Traumatol Arthrosc. (2015) 23(6):1770–81. 10.1007/s00167-014-3311-z 25218579

[39] Teel AJ Esposito JG Lanting BA Howard JL Schemitsch EH. Patellar resurfacing in primary total knee arthroplasty: a meta-analysis of randomized controlled trials. J Arthroplasty. (2019) 34(12):3124–32. 10.1016/j.arth.2019.07.019 31427130

